# Energy Harvesting Using Optimized ZnO Polymer Nanocomposite-Based 3D-Printed Lattice Structure

**DOI:** 10.3390/polym16212967

**Published:** 2024-10-23

**Authors:** Muni Raj Maurya, Mazen Alhamdi, Fawziya Al-Darwish, Faisal Sadek, Yousef Douglas, Nawar Karabili, Allaa Eltayeb, Roohollah Bagherzadeh, Shabi Abbas Zaidi, Kishor Kumar Sadasivuni

**Affiliations:** 1 Center for Advanced Materials, Qatar University, Doha P.O. Box 2713, Qatar; muni.raj@qu.edu.qa (M.R.M.); ma2003038@student.qu.edu.qa (M.A.); 2 Department of Civil and Environmental Engineering, Qatar University, Doha P.O. Box 2713, Qatar; 3 Department of Mechanical and Industrial Engineering, Qatar University, Doha P.O. Box 2713, Qatar; fa2301916@student.qu.edu.qa (F.A.-D.); fs2208113@qu.edu.qa (F.S.); yd2203608@qu.edu.qa (Y.D.);; 4 Department of Electrical Engineering, Qatar University, Doha P.O. Box 2713, Qatar; ae2204564@qu.edu.qa; 5 Advanced Fibrous Materials LAB (AFM-LAB), Institute for Advanced Textile Materials and Technologies (ATMT), Amirkabir University of Technology (Tehran Polytechnic), Tehran 1591634311, Iran; bagherzadeh_r@aut.ac.ir; 6 Department of Chemistry and Earth Sciences, College of Arts and Sciences, Qatar University, Doha P.O. Box 2713, Qatar

**Keywords:** three-dimensional printing, photopolymerization, energy harvesting, ZnO nanorods, nanogenerator

## Abstract

A 3D-printable polymer can provide an effective solution for developing piezoelectric structures. However, their nanocomposite formulation and 3D printing processability must be optimized for fabricating complex geometries with high printability. In the present study, we optimized the 3D-printable piezoelectric composite formulation for developing complex geometries by an additive manufacturing approach. The zinc oxide (ZnO) nanomaterial was synthesized by the hydrothermal method. The ZnO loading in the 3D-printed flexible resin was optimized to exhibit good interfacial adhesion and enable 3D printing. The lattice structure was fabricated to improve the piezoelectric response compared with the solid structure. The lattice structure block printed with 10 wt% ZnO showed a good piezoelectric response, with a linear increase in the generated output voltage for an increase in force. The maximum power density of 0.065 μW/cm^2^ was obtained under 12 N force at 1 Hz. The fabricated structure generated a peak–peak voltage of ~3 V with a foot heel strike.

## 1. Introduction

Mechanical stimuli in the immediate environment include human motion, waves, rain, and wind. Utilizing these stimuli from the surroundings to generate electricity has emerged as a competitive solution for renewable-based energy harvesting [1,2]. In recent times, there has been significant interest in using piezoelectric energy generators to supply electricity to the internet-of-things, wearable technology, and energy harvesting [3,4,5]. Piezoelectric materials possess the unique ability to convert mechanical stimuli into electrical energy and vice versa, and have found wide-ranging applications in sensors [4], actuators [6], energy harvesting devices [7], and biomedical devices [8]. In recent years, polymeric materials based on piezoelectric [9], flexoelectric [10], triboelectric [11], and hybrid systems have been appealing due to their ease of processing and flexibility, which make them well-suited for harvesting mechanical energy across various scales of mechanical stimuli. Different materials such as single crystals [12], metal oxides [13], 2D materials [14], carbon [15], and their composites are loaded in the polymer to fabricate flexible structures. Nanomaterial loading optimization in soft matter is crucial for enhancing the functionality of flexible devices and adjusting the physical properties of designs that are made of soft substrates. However, traditional manufacturing methods often pose material complexity and geometric intricacy limitations. Nanomaterial-loaded 3D-printed polymeric geometries can be a promising solution, where loaded nanomaterials exert piezoelectricity, printed geometries provide rigidity to the structure, and polymer bases provide flexibility for the extended area [16]. The emergence of 3D printing has overcome many of these challenges, offering unprecedented flexibility in material design and structural fabrication [17]. The amalgamation of 3D printing technology with piezoelectric materials has raised considerable interest in recent years, promising advancements in the domain of electromechanical systems. By adopting additive manufacturing processes, researchers have unlocked new opportunities for engineering piezoelectric composites with tailored properties and intricate architectures. Three-dimensional printing offers numerous advantages, such as flexibility in designing complex 3D architectures, high-end automation, scalability, and a minimum waste of material. Among different types of 3D printing techniques, digital light processing (DLP) can fabricate complex structures with high resolution and rapid production speed [18]. Although polymeric structures are being constructed using the DLP technique, the process of developing nanomaterial-polymer piezoelectric structures using DLP printing is challenging. The piezoelectric properties of 3D-printed polymer nanocomposites depend heavily on their functionality and processability. Poor compatibility and dispersion in printed composites can severely degrade piezoelectric performance due to weak interfacial bonding and inefficient transfer between the polymer matrix and the nanoparticles [19,20]. Moreover, a high nanoparticle content can lead to increased scattering [21], particle agglomeration [22], and higher viscosity [23,24], adversely affecting printing accuracy. Several studies have also explored how dispersion influences the precision of printing [25,26]. Another challenge in printing piezoelectric structures arises from the mismatch between the high stiffness of the nanoparticles and the lower stiffness of the polymer matrix, which leads to poor interfacial adhesion [27]. This mismatch reduces the stress transfer efficiency from the polymer matrix to the piezoelectric inclusions, thereby limiting the material’s functional performance. Although increasing the stiffness of the matrix has been shown to enhance the piezoelectric response [27,28], it remains uncertain whether highly responsive and flexible piezoelectric materials can be achieved.

Thus, the optimization of nanomaterial loading in polymer resin is required to attain good interfacial adhesion during printing and piezoelectric response. Additionally, the piezoelectricity of the system can be tailored by nanomaterial morphology and the geometry of the fabricated device, offering good electromechanical responsiveness with a high surface area. Regarding structural design, complex 3D architectures with enhanced applied stresses can improve the piezoelectric response. Among these, 3D lattice structures fabricated with flexible materials offering flexibility and rigidity could be realized for piezoelectric applications.

Herein, we present a unique strategy to adjust the nanomaterial loading and fabricate a piezoelectric structure using optimal 3D printing. The research methodology is based on generating structural design, configuring printing resin composition, and optimizing print settings for fabricating 3D geometries using a DLP 3D printer. We demonstrate that the as-fabricated lattice-structured sample has a piezoelectric voltage output and high stability originating from effective interfacial adhesion. The fabricated piezoelectric sample application was demonstrated by energy harvesting through human mechanical stimuli like foot strikes and tapping.

## 2. Materials and Methods

### 2.1. Chemicals and Reagents

Zinc acetate dihydrate (Zn(CH_3_COO)_2_·2H_2_O) (98%, CAS Number: 5970-45-6) and sodium hydroxide pellets (NaOH) (98%, CAS Number: 1310-73-2) were obtained from the Merck KGaA, Darmstadt, Germany. Flexible 3D printing resin Wanhao 61 Rubber (P/N: X60010202001W) was procured from Wanhao, Zhejiang, China. The chemicals used were all reagent grade without further purification before utilization. The experiments were conducted utilizing Millipore Milli-Q double-distilled water (Thermo Fisher Scientific Inc., Waltham, MA, USA).

### 2.2. Synthesis of ZnO

The hydrothermal method was used to synthesize the ZnO nanomaterial. A total of 5 g of zinc acetate dihydrate was dissolved in 100 mL of double-distilled water and stirred for 20 min. Separately, 3.75 g of NaOH was dissolved in 100 mL of double-distilled water and stirred for 15 min. The NaOH solution was then added dropwise into the aqueous zinc acetate solution under vigorous stirring for 30 min at 60 °C, forming a white, gelatinous precipitate. The pH was maintained at 9 pH. The obtained mixture was sealed in an autoclave, and hydrothermal synthesis was carried out at 180 °C for 6 h. The resulting nanocomposite was centrifuged and washed thoroughly with distilled water and ethanol five times to eliminate any residue. Finally, the product was dried at 80 °C for 1 h, followed by annealing at 400 °C for 4 h.

### 2.3. Three-Dimensional Printing of ZnO-Loaded Lattice Structures

**Figure 1:**
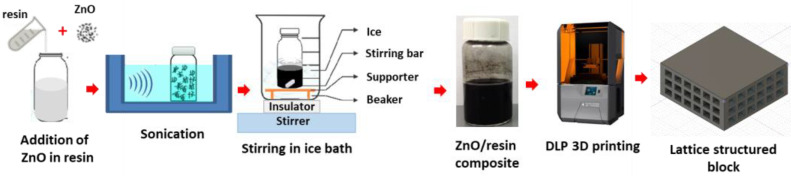
Schematic representing ZnO loading in resin and 3D printing.

The solid and lattice-structured blocks were fabricated separately using a DLP 3D printer (Phrozen Sonic Mini 8K resin 3D printer, Hsinchu, Taiwan). Following the preparation of the ZnO composite mixture as previously described, resin loaded with the 8 wt%, 10 wt%, and 12 wt% of ZnO was prepared by the solution mixing method, as shown in Figure 1. The known weight percentage of ZnO was added to 20 g of resin and sonicated in ice water for 30 min. The obtained ZnO-mixed resin was further stirred for 2 h, and the temperature was lowered below 5 °C using ice water. Both sonication and stirring were performed under dark conditions to avoid any curing of the resin under light. The obtained ZnO-loaded resin was used for the 3D printing of the lattice-structured and solid blocks. The 3D printing parameters are listed in Table 1.

### 2.4. Characterization

The structure of the ZnO was examined with an X-ray diffractometer (X’PERT-Pro MPD, PANalytical Co., Almelo, The Netherlands). The ZnO structure was confirmed with a thermo Nicolet Nexus 670 FTIR spectrometer (KBr pellets) (Thermo Fisher Scientific Inc., Waltham, MA, USA). The morphologies of the ZnO and the 3D-printed block were studied by scanning electron microscopy (SEM, Hitachi S-4800, Hitachi, Tokyo, Japan). Energy-dispersive X-ray analysis (EDAX) was used to identify the elements in the nanomaterial. A RSA-G2 dynamic mechanical analyzer with a maximum force of 35 N was used to apply the variable force at different frequencies. The fabricated samples were prepared in sizes of 2 × 2 cm^2^, and silver paste was applied to the top and bottom surfaces to serve as an electrode. Copper wire was attached to the silver paste, and the electrode surface was covered with Kapton tape (Ted Pella Inc., Redding, CA, USA). All measurements were performed on an effective area of 4 cm^2^. Simultaneously, the open circuit voltage was studied by acquiring the output voltage under varied forces using the national instrument NI USB-4431 (National Instruments Corp., Austin, TX, USA). The short circuit current and current under load were measured by a Keithley 2400 (Tektronix Inc, Cedar Hills, OR, USA).

## 3. Results

### 3.1. Microstructural Characteristics

**Figure 2:**
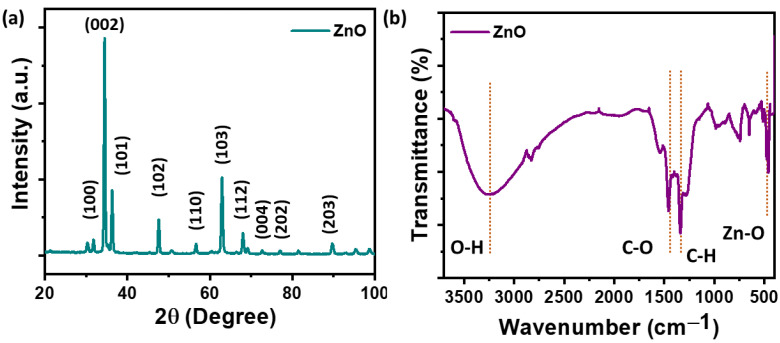
Structural analysis of the synthesized ZnO nanomaterial. (**a**) XRD plot of the ZnO. (**b**) FTIR spectra of the ZnO. The red dotted vertical lines indicate the peak assigned to the function group.

The structure of the synthesized nanomaterial was analyzed using X-ray diffraction (XRD). Figure 2a illustrates the XRD spectra for pure ZnO synthesized via the hydrothermal method. The observed XRD peaks at 2θ values of 31.77°, 34.47°, 36.27°, 47.57°, 56.62°, 62.97°, 68.62°, 72.72°, 76.97°, and 89.62° corresponded to the (100), (002), (101), (102), (110), (103), (112), (004), (202), and (203) planes, respectively. The XRD data confirm the hexagonal wurtzite structure of the ZnO, in agreement with standard data (JCPDS-36-1451) [29]. Notably, all samples displayed a high orientation along the (002) reflection plane, indicating the favorable vertical growth of the ZnO along the c-axis. The vibration behavior of the synthesized nanomaterial was analyzed by FTIR spectroscopy. The typical infrared plot of the ZnO is presented in Figure 2b. A broad band near 3000–3800 cm^−1^ with a peak centered at nearly 3450 cm^−1^ is attributed to the O-H group stretching on the surface of the ZnO [30]. Peaks between 400 cm^−1^ and 550 cm^−1^ represent the metal–oxygen stretching mode. In the FTIR spectra of the ZnO, a peak at 447 cm^−1^ is observed, corresponding to the Zn-O stretching [30].

### 3.2. Morphology Study

**Figure 3:**
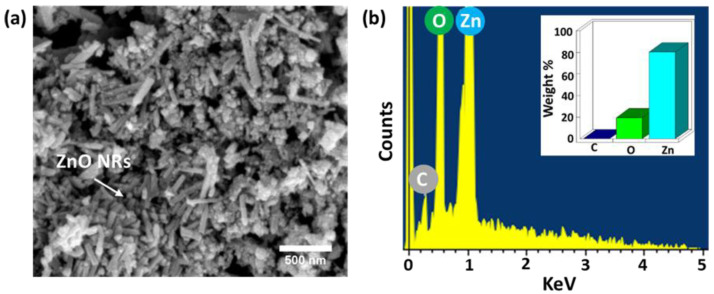
Morphology analysis of ZnO. (**a**) SEM image of ZnO confirming the growth of NRs. (**b**) EDAX plot of ZnO. The inset in the figure shows the weight percentages of the elements in the ZnO.

The morphology of the synthesized ZnO was examined by SEM analysis. Figure 3a shows the SEM image of the synthesized ZnO. The formation of the ZnO nanorods (NRs) was confirmed by the SEM analysis. The NR growth is in agreement with the XRD data, with the dominant (002) plane peak confirming vertical growth along the c-axis. The elements in the ZnO sample were examined with the EDAX. Figure 3b shows the EDAX plot of the ZnO sample. The presence of Zn and O peaks in the EDAX confirms the successful formation of the ZnO and supports the XRD/FTIR study. The inset in Figure 3b shows the weight percentages of the elements in the sample. The carbon peak in EDAX comes from the carbon tape used as a substrate to keep the ZnO sample during the SEM analysis.

**Figure 4:**
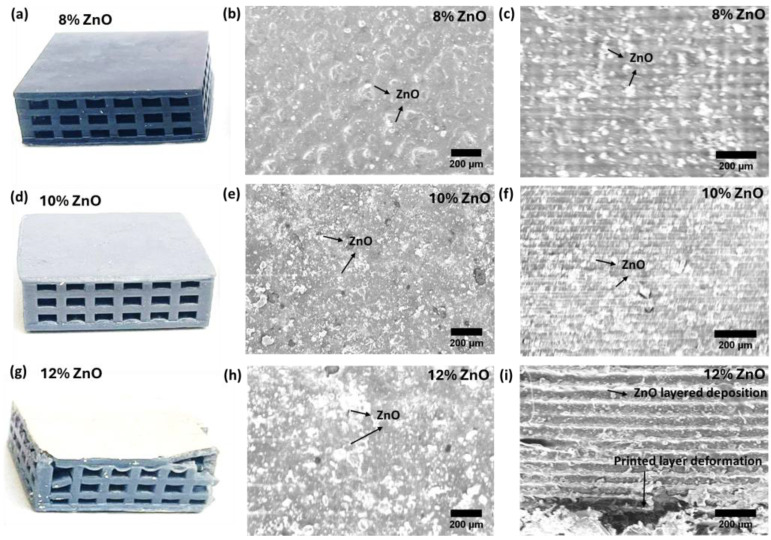
The structure analysis of the 3D-printed lattice block. (**a**) A picture of the sample printed with 8 wt% ZnO loading in resin. (**b**) A surface SEM image of the lattice block, 3D-printed with 8 wt% of ZnO. (**c**) A cross-sectional SEM image of the 8 wt% ZnO sample. (**d**) A photograph of the sample printed with 10 wt% ZnO loading in resin. (**e**) A surface SEM image of the lattice block, 3D-printed with 10 wt% of ZnO. (**f**) A cross-sectional SEM image of the 10 wt% ZnO sample. (**g**) A picture of the lattice block, 3D-printed with 12 wt% of ZnO. (**h**) A surface SEM image of the lattice block, 3D-printed with 12 wt% of ZnO. (**i**) A cross-sectional SEM image of the 12 wt% ZnO sample.

Figure 4 shows the photograph and SEM image of the 3D-printed lattice-structured block with its resin loaded with different weight percentages of the ZnO nanomaterial. The picture of the lattice block 3D printed with 8 wt% ZnO is shown in Figure 4a. The structure displayed a compact layer architecture without detachment. Figure 4b,c show the surface and cross-sectional SEM of the sample printed with 8 wt% ZnO-loaded resin, respectively. The surface SEM image displays the dispersion of ZnO and indicates that the nanoparticles tended to accumulate and form isolated islands at the loading concentration of 8 wt%. The cross-section image of the sample in Figure 4c shows horizontal lines that correspond to the layers of the printed structure. The SEM image displays compact bonding between layers. Figure 4d presents the picture of a lattice-structured block printed with 10 wt% ZnO-loaded resin. The surface SEM image of the 10 wt% ZnO sample displays an increase in the coverage area of the ZnO, indicating the formation of an interconnected ZnO island network in the polymer matrix, as shown in Figure 4e. The cross-sectional SEM image of the 10 wt% ZnO sample exhibits a compact layer-by-layer printed architecture, and the bright region in the image represents the formation of an interconnected ZnO network within the resin matrix. However, with an increase in the ZnO loading percentage to 12 wt%, the printed lattice structure displayed detachment between the layers of the structure, as shown in Figure 4g. The surface SEM image of the 12 wt% ZnO-loaded sample displays increased coverage of ZnO nanoparticles on the surface, indicating the formation of a ZnO layer on the surface of the resin (see Figure 4h). The formation of a ZnO layer on the surface indicates the settling of ZnO nanomaterial at the bottom of the resin tank. As a result, the interfacial bonding between the layers decreased, and the structure exhibited detachments in the printed layers, as shown in Figure 4i. With a further increase in the ZnO loading to 15 wt%, the settling of the ZnO nanomaterial in the resin tank significantly increased, and the structure was printed up to a few layers only. Moreover, the increase in ZnO loading to 15 wt% could also enhance the light scattering from ZnO, which could impact the curing of resin and result in layer detachment and no 3D printing.

### 3.3. Piezoelectric Response of Nanomaterial Loaded 3D-Printed Structure

**Figure 5:**
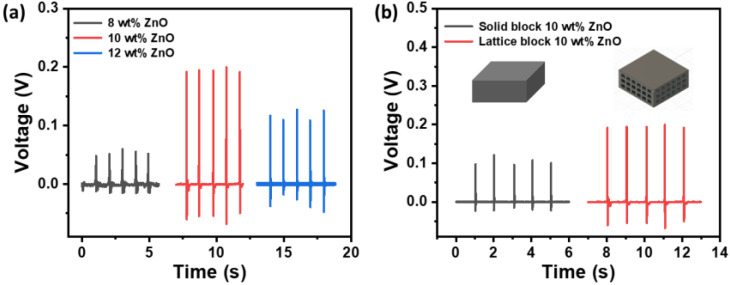
The piezoelectric response of the 3D-printed blocks with an effective area of 4 cm^2^. (**a**) The open circuit output voltage of the lattice-structured 3D-printed blocks with different weight percentages of ZnO loading under a 3 N force and a 1 Hz frequency. (**b**) The open circuit output voltage of the lattice-structured and solid 3D-printed blocks with 10 wt% of ZnO under a 3 N force and a 1 Hz frequency.

The energy harvesting performance and its correlation with ZnO loading in resin and 3D-printed structure architecture were examined for piezoelectric power generation, as presented in Figure 5. Open circuit output voltage was measured under a 3 N force and a 1 Hz frequency with an effective area of 4 cm^2^. For the lattice-structured block, the generated output voltage increased with increases in ZnO loading up to 10 wt%. However, with a further increase to 12 wt% of ZnO loading, a decrease in open circuit voltage was observed, as shown in Figure 5a. The decrease in the output voltage of the 12 wt% ZnO sample could have been due to the detachment of layers, as observed in the SEM image (see Figure 4c), leading to a loss in charge transfer. Compared to the 8 wt% and 12 wt% ZnO-loaded samples, the 10 wt% ZnO sample exhibited high output voltage. To analyze the impact of architecture, the output voltage was measured from a 10 wt% ZnO-loaded solid block and a lattice-structured block under 3 N force and 1 Hz frequency. The lattice-structured block displayed improved performance with a peak-to-peak open circuit voltage (V_pp_) of 0.26 V, compared to the solid block with its V_pp_ of 0.14 V. Two attributes can be considered for lattice structure improvement of the nanogenerator output. The first is the greater ability to transfer mechanical energy into an electrical signal of the lattice structure compared to the solid block. The second active mechanism, which increased the output, could be attributed to the reduced volume of polymer resin, which reduces the charge loss of the ZnO polymer matrix and increases the collection of charges under the same force. This means that the samples with lattice structures had higher potentials to deliver the excited charges to the electrodes to be collected at the surface. Thus, the lattice architecture substantially impacts the output voltage and improves energy harvesting. The 10 wt% ZnO-loaded lattice-structured block was considered for further energy harvesting studies.

### 3.4. Piezoelectric Responses of the 10 wt% ZnO Loaded Lattice-Structured Block

**Figure 6:**
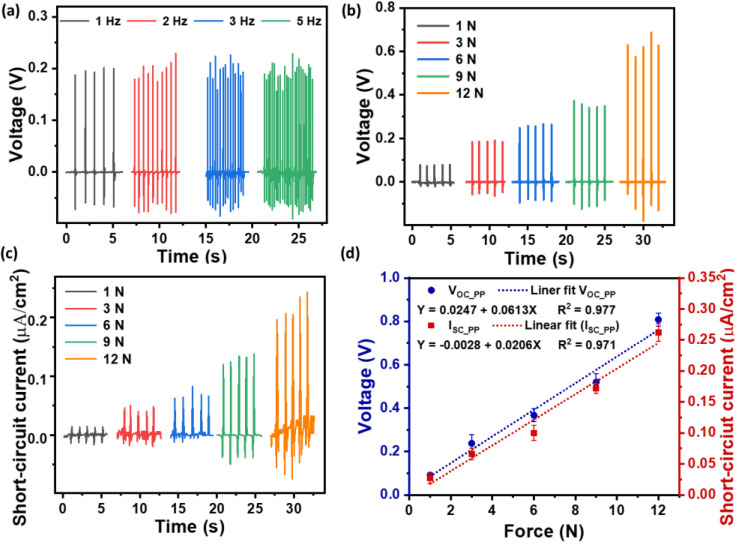
The piezoelectric response of the 3D-printed 10 wt% ZnO-loaded lattice-structured block with an effective area of 4 cm^2^. (**a**) The open circuit output voltage under different frequencies and 3 N of force. (**b**) The output voltage under varying forces at 1 Hz. (**c**) The short circuit current density under varying force at 1 Hz. (**d**) Linear relationships of peak-to-peak piezoelectric voltage and short circuit current density with varying loads under 1 Hz.

The investigation focused on evaluating the piezoelectric performance of the 10 wt% ZnO-loaded lattice-structured block. The output voltage from the lattice block was measured under variable frequencies (1 Hz, 2 Hz, 3 Hz, and 5 Hz), and 3 N force. The sample exhibited a similar output voltage response with peak-to-peak voltage response varying between 0.26 and0.3 V, as shown in Figure 6a. Thus, for further study, a 1 Hz frequency was considered for piezoelectric analysis. Figure 6b shows the generated output voltage under varied forces (1 N, 3 N, 6 N, 9 N, and 12 N) at 1 Hz. A substantial increase in output voltage was obtained with an increase in the applied force. The measured short circuit current density result under varied forces (1 N, 3 N, 6 N, 9 N, and 12 N) at 1 Hz is presented in Figure 6c. The measured current (I_sc_) also exhibited an increased current with an increase in applied force. Figure 6d summarizes the piezoelectric peak-to-peak open circuit voltage (V_oc_pp_) and peak-to-peak short circuit current density (I_sc_pp_) measured under varied force at 1 Hz. The piezoelectric voltage and current increment with increased force were linearly fitted and obtained a good linear fit with an R^2^ value of ~0.97, suggesting a consistent response to varied mechanical stresses. The mechanical sensitivity of the 3D-printed lattice block was found to be 0.06 V/N, which is an encouraging result for applications requiring sensitivity to mechanical stimuli.

**Figure 7:**
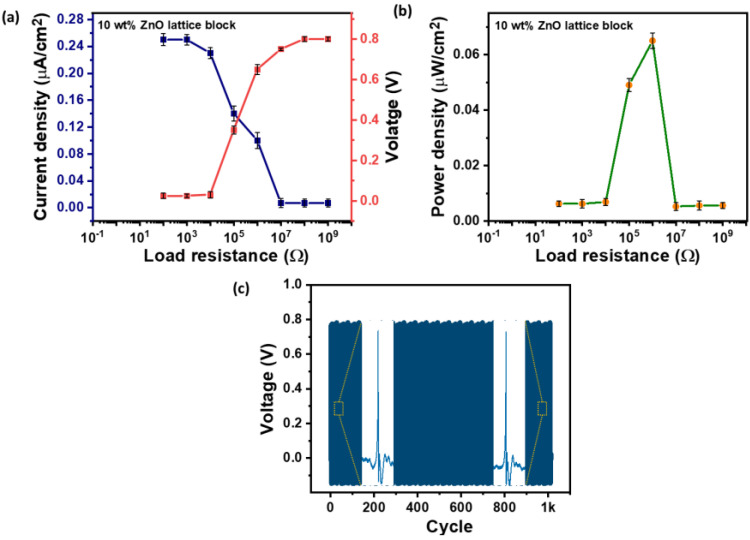
(**a**) Voltage and current density versus load resistance for the 10 wt% ZnO-loaded lattice block under 6 N and 1 Hz. (**b**) Power density versus load resistance. (**c**) Output voltage for 1000 cycles of loading and unloading of 6 N force at 1 Hz. The yellow-outlined square represents the zoomed-in image of the generated piezoelectric voltage signal during the initial of final force cycles.

The output current density and voltage generation demonstrate the device’s energy-harvesting capability, confirming the effectiveness of the material system, structural design, and 3D printing process. Voltage and current outputs were measured under varying load resistances (Figure 7a). As expected from Ohm’s law, the output voltage increased while the current density decreased with higher load resistance. Power density was calculated by multiplying voltage and current density. Figure 7b shows the power density relationship with the load resistance. The maximum power density of 0.065 μW/cm^2^ was achieved at a load of 1 MΩ.

**Figure 8:**
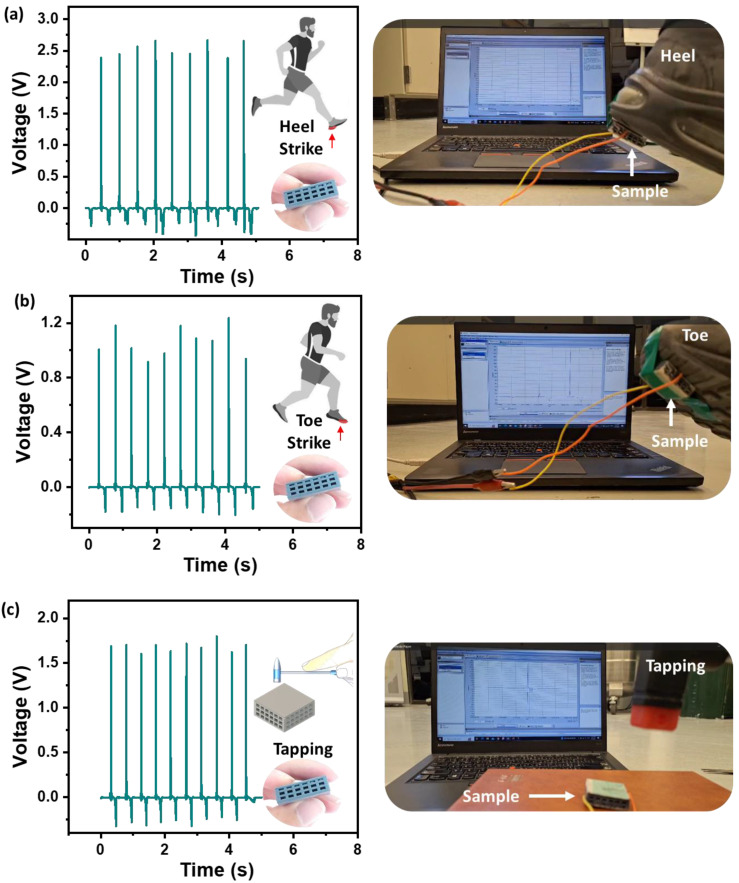
The output voltage generated by mechanical stimuli. (**a**) The voltage performance of the lattice block under foot heel strike. (**b**) The voltage generated under foot toe strike. (**c**) The output voltage under tapping.

The reported piezoelectric structure’s output power capabilities and high weight-bearing efficiency could be applied to energy harvesting from mechanical motion. The flexible 3D system can act as a smart system, generating output voltage under different mechanical conditions. Figure 8 demonstrates the device’s effectiveness as a body-integrated energy harvester. The 3D-printed lattice sample was attached under the shoe sole and tested against human movements to evaluate its energy-harvesting potential. When integrated into footwear, the sample was able to capture energy from steps, generating approximately 3 V from heel strikes (Figure 8a) and around 1.4 V from toe strikes (Figure 8b). The picture of the heel and toe strike is shown in Figure 8a (right) and Figure 8b (right), respectively. The voltage signal generated during heel and toe strikes is shown in Supplementary Videos S1 and S2, respectively. This application is promising for use in fitness-tracking insoles and self-powered smart shoes. The sample tapping generated ~ 2V, as shown in Figure 8c. The picture and video of the sample tapping are shown in Figure 8c (right) and Supplementary Video S3, respectively. The sample maintained its structural integrity throughout these experiments, which is vital for its application in energy harvesting tiles, racing/jogging tracks, and developing play areas for sports like badminton, volleyball, basketball, etc., and could also power the sensors used in these games.

## 4. Conclusions

In summary, the 3D-printed lattice structure with a piezoelectric–polymer composite was fabricated using DLP printing technology. First, the ZnO nanorods (NRs) were synthesized by the hydrothermal method. The structure and morphology were studied using XRD and SEM analysis, respectively. The formulation of the ZnO polymer was obtained by loading different weight percentages of ZnO. Second, the lattice structure was designed and printed for 8 wt%, 10 wt%, and 12 wt% of ZnO loading. Samples up to 10 wt% ZnO loading displayed good dispersion stability, interfacial adhesion, and printability. The lattice structure sample improved the piezoelectric response compared to the solid block, and the 10 wt% ZnO-loaded lattice sample displayed the highest output voltage. Printing and improvements in energy harvesting were analyzed through SEM images of samples. The piezoelectric response of the 10 wt% ZnO lattice sample was 0.83 V under a 12 N load at 1Hz. Both the generated peak-to-peak open circuit voltage and short circuit current displayed linear proportionality with force with a mechanical sensitivity of 0.06 V/N. The 3D-printed lattice block with 10 wt% ZnO loading improved the piezoelectric responses and obtained a power density of 0.065 μW/cm^2^ when the load was 1 MΩ under 12 N at 1 HZ. This study highlights that 3D-printed piezoelectric composites with complex designs like lattice structures can be employed for energy harvesting using human, mechanical stimuli. The foot heel strike exhibited an output voltage of ~3 V. This study has applications in energy harvesting, mechanical stimuli monitoring systems, wearable electronics, and devices.

## Figures and Tables

**Table 1:** Three-dimensional printing parameters.

3D Printing Parameters	Condition
Layer height (mm)	0.05
Bottom layer exposure time (s)	50
Layer exposure time (s)	8
Lift speed (mm/min)	60
Lift distance (mm)	5
Retract speed (mm/min)	150

## Data Availability

The original contributions presented in the study are included in the article/Supplementary Materials; further inquiries can be directed to the corresponding author/s.

## Supplementary Materials

The following supporting information can be downloaded at: https://www.mdpi.com/article/10.3390/polym16212967/s1; Video S1: Energy harvesting with heel strike; Video S2: Energy harvesting with toe strike; Video S3: Energy harvesting with tapping.
